# An imbalance data quality monitoring based on SMOTE-XGBOOST supported by edge computing

**DOI:** 10.1038/s41598-024-60600-x

**Published:** 2024-05-02

**Authors:** Yan Han, Zhe Wei, Guotian Huang

**Affiliations:** 1 School of Mechanical Engineering, Shenyang University of Technology, Shenyang, 110870 China; 2 Genertec Shenyang Machine Tool Co., Ltd, Shenyang, 110041 China

**Keywords:** Aerospace engineering, Mechanical engineering

## Abstract

Product assembly involves extensive production data that is characterized by high dimensionality, multiple samples, and data imbalance. The article proposes an edge computing-based framework for monitoring product assembly quality in industrial Internet of Things. Edge computing technology relieves the pressure of aggregating enormous amounts of data to cloud center for processing. To address the problem of data imbalance, we compared five sampling methods: Borderline SMOTE, Random Downsampling, Random Upsampling, SMOTE, and ADASYN. Finally, the quality monitoring model SMOTE-XGBoost is proposed, and the hyperparameters of the model are optimized by using the Grid Search method. The proposed framework and quality control methodology were applied to an assembly line of IGBT modules for the traction system, and the validity of the model was experimentally verified.

## Introduction

With the vigorous promotion of Industry 4.0 and Intelligent Manufacturing, manufacturing systems are rapidly integrating with new technologies such as the Internet of Things (IoT), Artificial Intelligence (AI), and Machine Learning (ML). In a smart manufacturing environment, the manufacturing equipment in an intelligent factory is equipped with numerous sensors that generate data with “3V” characteristics, namely Volume, Velocity, and Variety. These data contain valuable information about the manufacturing. Utilizing real-time data generated by intelligent devices for product quality monitoring has become a crucial objective in the field of intelligent manufacturing^1^. In the context of intelligent manufacturing, the powerful computing and storage capabilities of cloud computing centers are widely acknowledged. Various data analysis tasks are deployed to the cloud centers. However, there are still some challenges. The network transmission bandwidth may experience an increase during the process of uploading a substantial volume of data to the cloud center, resulting in network latency.

Edge computing, as a complementary computing approach to cloud computing, is a novel distributed computing method that enables processing at the edge of the internet^2^. In comparison to cloud computing, edge computing offers shorter response times and higher reliability. It has been extensively utilized in various domains, including smart cities^3^, smart transportation^4,5^, and other industrial areas^6–8^. Usman et al.^9^ proposed a multilevel edge computing framework named as the Reliability and Security Edge Computing (RaSEC) intelligent framework. Zhao et al.^10^ introduced a heuristic algorithm that utilizes edge computing to tackle data processing challenges in smart grids, resulting in significant reductions in data transmission times. Qian et al.^11^ conducted a study on the utilization of edge computing for real-time fault diagnosis and dynamic control of rotating machinery. Their research demonstrated a substantial enhancement in the speed and accuracy of fault detection. Blanco-Novoa et al.^12^ assigned computational tasks within the system to edge computing, enhancing the real-time responsiveness in intelligent manufacturing systems. Atan et al.^13^ proposed an AI-driven approach for efficiently executing tasks in heterogeneous IoT applications. This approach effectively reduces decision latency and enhances real-time performance by incorporating edge computing technology. Liang et al.^14^ employed edge computing technology to transfer the training process of deep learning models from the cloud to edge nodes, thereby alleviating network congestion. Wang et al.^15^ proposed a novel method for data collection and cleaning that utilizes mobile edge nodes. In conclusion, edge computing has progressively emerged as a pivotal factor in driving the digital transformation of smart manufacturing, effectively expediting the modernization of the manufacturing industry.

As the manufacturing industry progresses towards higher levels of intelligence, the monitoring of product quality during the production processes becomes increasingly crucial. Early identification and implementation of preventive measures against quality issues can lead to cost reduction and improve production efficiency. During the manufacturing processes, potential issues with the quality of the product can be efficiently identified by conducting thorough analysis of the production data in order to monitor product quality. The introduction of artificial intelligence technology offers new opportunities for data-driven monitoring of the production processes. These involve the use of intelligent learning methods such as Artificial Neural Network (ANN)^16^, Support Vector Machine (SVM)^17^, Fuzzy Logic^18^, and the integration of various intelligent approaches^19^. Chen et al.^20^ proposed a method for surface roughness detection using a “multi-dimensional feature parameter matrix + BP neural network algorithm + automatic acquisition of the region of interest”. Ren et al.^21^ introduced a data-driven approach for intelligent industrial production processes using a Wide and Deep Sequence (WDS) model. This model was specifically designed to address the challenges posed by multi-source heterogeneous industrial data. Zhou et al.^22^ proposed a compact and computationally efficient algorithm called the Improved Orthogonal Incremental Random Vector Function Linkage Network (I-OI-RVFLN). They applied this algorithm to the quality modelling of the blast furnace ironmaking processes. Zhang et al.^23^ proposed a novel method for monitoring the quality of multi-unit industrial processes.

The monitoring of product quality during manufacturing processes has garnered significant attention from worldwide scholars; however, challenges still persist. In assembly lines, the number of conforming products far exceeds that of non-conforming products, resulting in an imbalance in data classification for quality monitoring. Additionally, the use of traditional cloud computing methods for product quality monitoring can lead to network congestion. To tackle these issues, this article proposes the utilization of edge computing techniques to alleviate the load on the cloud. Furthermore, the Synthetic Minority Oversampling Technique (SMOTE) is combined with extreme Gradient Boosting (XGBoost) to address the challenge of unbalanced data classification. Finally, the hyperparameters of the XGBoost algorithm are optimized to enhance the performance of the classification model.

The major contributions of this article are summarised as follows:

1. The article proposes an industrial Internet of Things (IoT) framework for assembly product quality monitoring. By leveraging the computational resources of edge devices, certain tasks offloaded from the cloud center to the edge for execution, effectively reducing the workload on the cloud center.
2. A quality monitoring method is proposed based on hyperparameter-optimized SMOTE-XGBoost_s for imbalanced data. This method provides an approach to address the problem of data imbalance theoretically and effectively improves the accuracy and efficiency of classification models in handling complex data in practical applications.

## A product assembly quality monitoring framework based on edge computing

**Figure 1:**
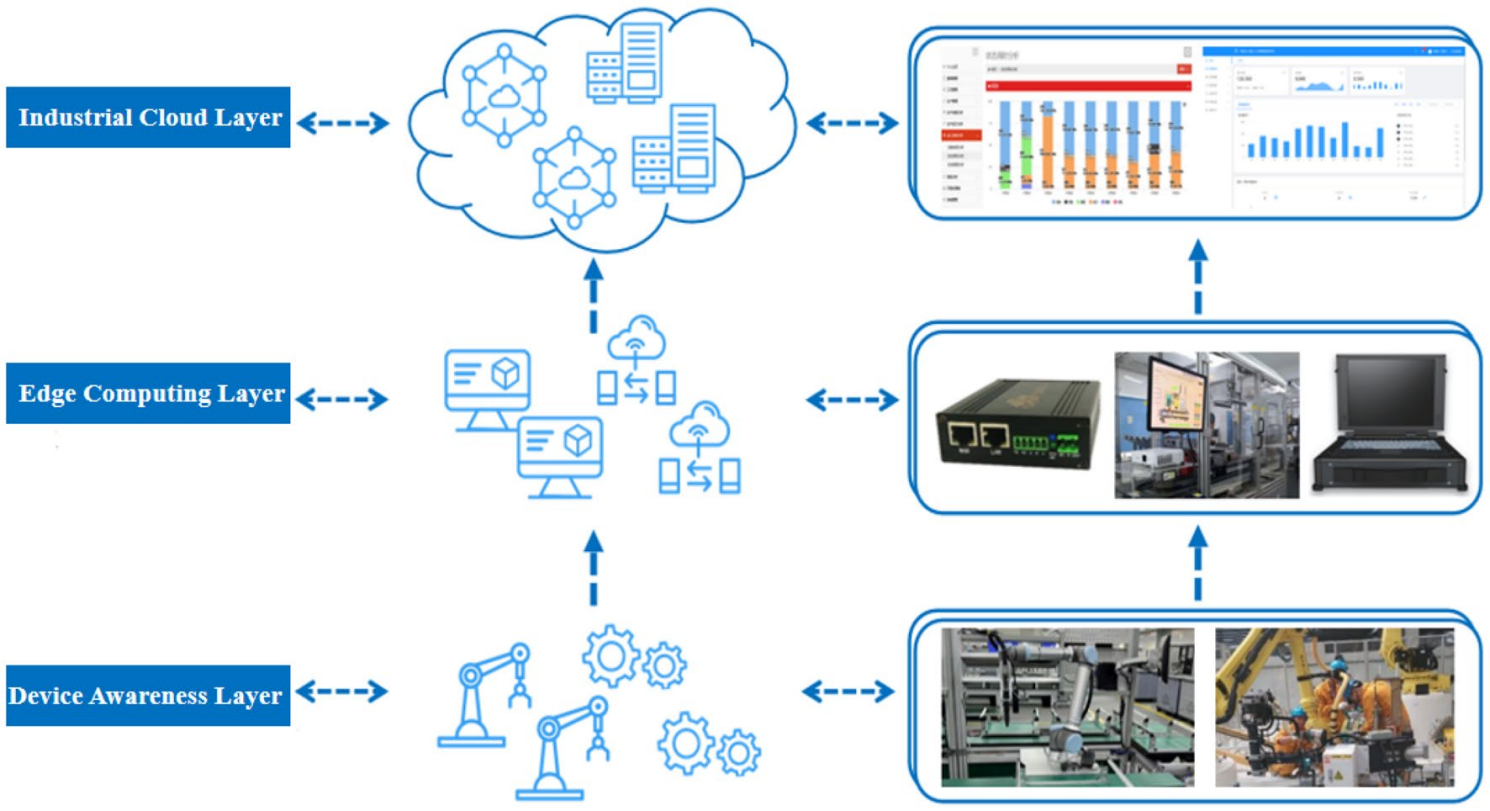
Industrial Internet of Things (IoT) framework for assembly product quality monitoring.

In industrial production settings, real-time monitoring of operations is of utmost importance. This practice facilitates the prompt tracking and analysis of production data, thereby enabling swift feedback and detection of faults. Traditional product quality monitoring methods have commonly employed a centralized processing architecture. The data is uploaded to the cloud center for analysis, computation, and storage. However, the process can lead to network latency due to the complexity of the processing steps involved. It can put a strain on cloud computing resources, resulting in a decrease in the overall stability of the system. In order to cope with these challenges, the decision was made to adopt edge computing technology. Edge computing offers significant advantages in various industrial scenarios, as it provides low latency and helps alleviate the burden on cloud computing infrastructures. The purpose of the article is to leverage the real-time response capabilities of edge computing technology and advanced computational techniques to develop an innovative intelligent framework and application method. The framework consists of three layers: the Device Awareness Layer, the Edge Computing Layer, and the Industrial Cloud Layer, as shown in Fig. 1.

### Industrial cloud layer

The industrial cloud layer consists of cloud computing servers, data servers, and web servers. Its main function is to establish connectivity with the edge computing layer in order to centrally manage the heterogeneous data of the edge computing layer. On the one hand, the layer is charged with the reception and storage of historical data, including computational results and alert situations. On the other hand, the layer utilizes Incremental Learning to update the existing product quality monitoring model and dispatches the model to the edge computing layer during periods of inactivity. At the same time, the industrial cloud layer also provides highly aggregated data analysis and storage service, including complex event processing, among others.

### Edge computing layer

The edge computing layer is positioned between the device awareness layer and the industrial cloud layer. It primarily consists of data transmission units, edge servers, gateways, and other devices. The main functions of the layer include data transmission, data pre-processing, data decision-making, and data computation. By utilizing the hardware resources at the edge of the production lines, it filters crucial information, conducts data format conversion, and pre-processes the data, effectively reducing the computational load on the cloud center. Meanwhile, the edge computing layer realizes the interconnection among various edge nodes and addresses the issue of “information island” in industrial production facilities.

### Device awareness layer

The device awareness layer comprises testing equipments, such as operating platforms and bolt tightening equipments, which gather data such as torque value, preload force, and part number. The device awareness layer not only encompasses real-time product status, but it also receives operational commands from the industrial cloud layer and the edge computing layer in order to achieve prompt responses from the terminal equipment.

## Smote_XGBoost quality monitoring method with hyper-parameter optimisation

**Figure 2:**
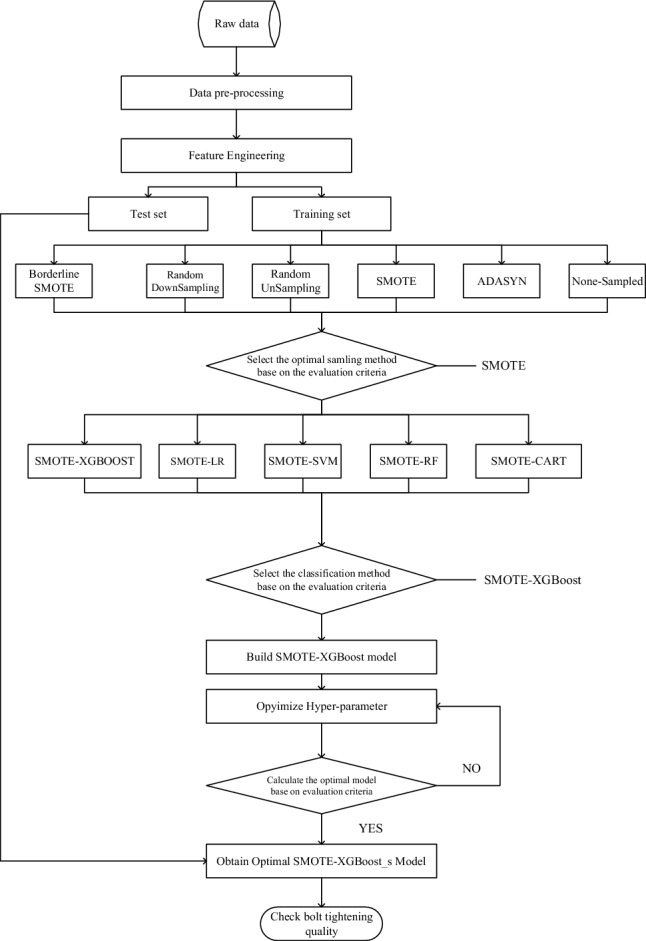
The flowchart of the assembly quality monitoring method.

In order to address the product quality issues of unbalanced data on the assembly line, we propose a product quality monitoring method. We preprocess and conduct feature engineering on the original data obtained from the production assembly line. We then utilize stratified sampling to partition the original dataset into a training set and a test set. The approach ensures that the distribution of categories in both the training and test sets is comparable to that of the original dataset. To address the issue of imbalanced data, we apply various resampling algorithms, namely Borderline SMOTE, Random Downsampling, Random Upsampling, SMOTE, and ADASYN, to the training set. Our objective is to achieve a balanced dataset. Finally, based on the previously processed balanced dataset, we have developed a SMOTE-XGBoost quality monitoring model. We conducted comparative experiments on hyperparameter optimization for the model using both grid search and random search methods. Measure the effectiveness of the model against the evaluation metrics and output the final results. The detailed process is shown in Fig. 2.

### Data pre-processing

**Figure 3:**
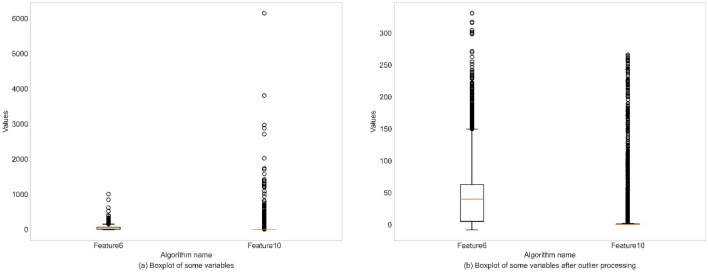
Data outlier handling.

The data presented in the article represents actual data that was collected from product assembly lines. The data is divided into historical data and real-time data. Historical data refers to the information collected and stored in the cloud by the edge computing layer, while real-time data refers to data directly gathered by the device awareness layer. The data processing procedure consists of three main steps: missing value processing, data type conversion, and outlier handling. The details are as follows:

1. Missing value processing: Data missing are termed as incomplete data. Missing values processing is crucial to prevent errors in subsequent models due to high missing rates. We eliminated Feature 9, which had a missing value weight exceeding 90%, thereby enhancing the model’s predictive accuracy.
2. Data type conversion: For the data in this article, we converted non-numeric type Feature2, Feature3, Feature4, and Feature11 as numeric type.
3. Outlier handling: Outliers are caused by sensor malfunctions, human input errors, or unusual events. In the data after type conversion, there were 7 outliers in Feature 6 and 102 outliers in Feature 10. This article addresses outliers by using a threshold method. Outliers below the set minimum threshold are overridden by the set minimum threshold, and those above the set maximum threshold are overridden by the set maximum threshold, as shown in Fig. 3.

### Feature engineering

**Figure 4:**
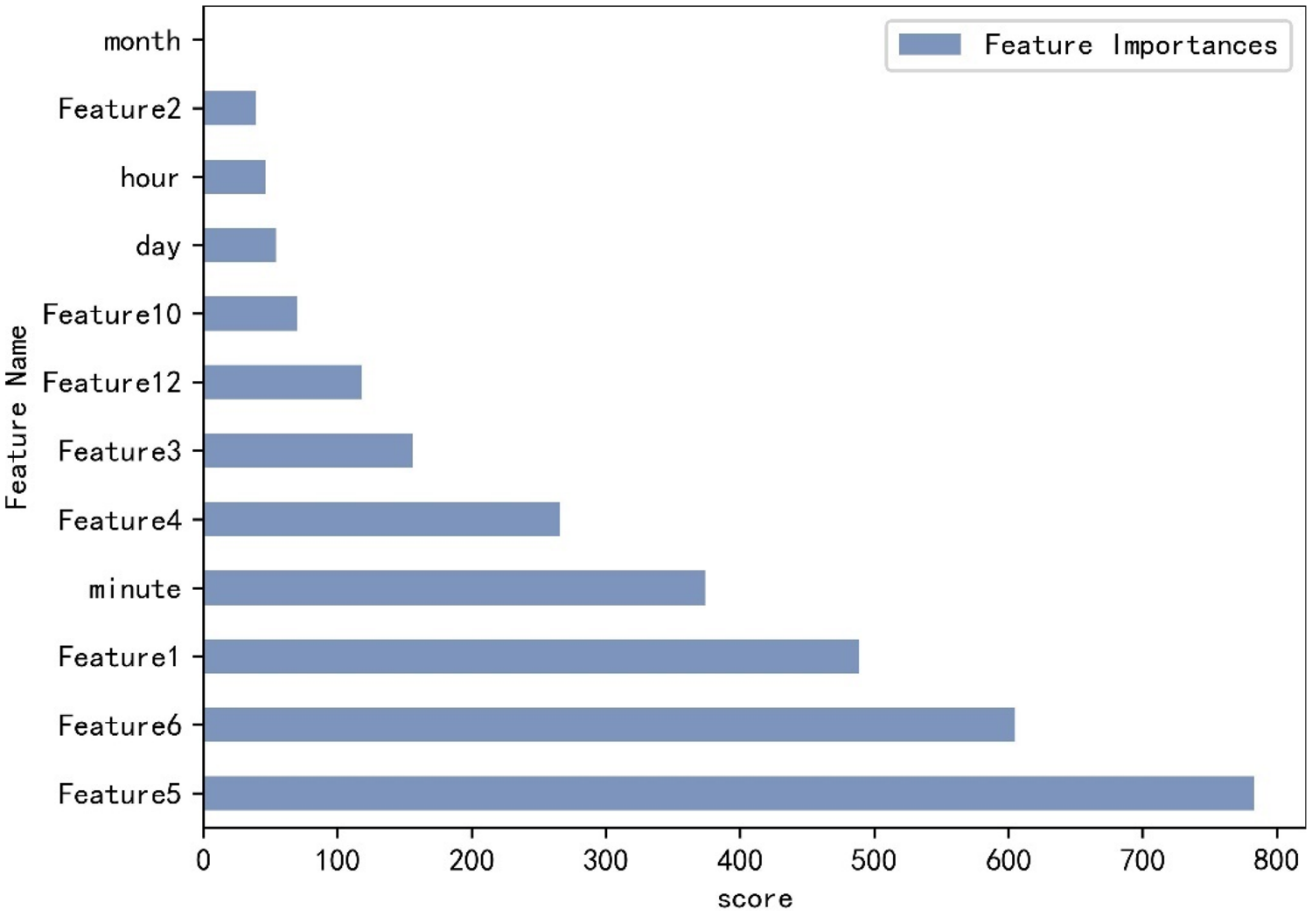
Feature importance arrange.

Feature engineering is a critical process in machine learning. It involves transforming pre-processed data into a format that is suitable for model training. Its primary purpose is to obtain better features, enabling machine learning models to maximize their potential with the data, thereby enhancing the performance of machine learning models. According to the data in this article, feature engineering is divided into feature construction and feature selection, as follows:

1. Feature Construction: Feature construction is a crucial part of feature engineering, which can significantly enhance the training efficiency and prediction accuracy of models. Upon observing the data, we need to reconstruct Feature14. Feature14 represents a time pattern, which we will divide into five new features: year, month, day, hour, and minute, and then remove the original Feature14.
2. Feature Selection: After preprocessing the data, analyzing and constructing the features, there may still be irrelevant and unnecessary features. To enhance model efficiency, reduce algorithm time, and simplify the learning process, feature selection is a crucial step. Initially, we eliminated Feature7, Feature8, and year because their variance values are 0. Then, it was discovered that Feature12 and Feature13 contained identical information. As a result, the redundant sequence of Feature13 was removed, while Feature12 was retained. The LightGBM is a Boosted Ensemble model developed by Microsoft in 2017. It is a high-accuracy and high-speed Gradient Boosting framework algorithm that transforms weak learners into potential models. The LightGBM algorithm is used to measure the feature importance, as shown in Fig. 4.

### Smote_XGBoost with assembly quality prediction

We employ the stratified sampling method to divide the imbalanced dataset: 70% for the training set and 30% for the test set.

Synthetic Minority Over-Sampling Technique algorithm (SMOTE) is an over-sampling algorithm proposed by Chawla and Bowyer et al. in 2002. Based on the Linear Interpolation method, it utilizes the existing minority class samples to synthesize new samples further to enhance the imbalanced dataset’s classification accuracy^24^. EXtreme Gradient Boosting algorithm(XGBoost)^25^ is an ensemble boosting algorithm based on the CART learning model and proposed by Dr Tianqi Chen in 2016. XGBoost is an improved model of the Gradient Boost Decision Tree algorithm(GBDT)^26^, and the objective function is composed of two parts: the Loss Function and the Regularize. The XGBoost algorithm expands the loss function to the second order Taylor series, and incorporates a regularization term into the optimization of the loss function as a whole. This approach balances the reduction of the loss function and the complexity of the model, thus avoiding overfitting and enhancing the model’s generalization ability. The algorithm efficiently utilizes CPU multi-threading for parallel computation, characterized by fast training speed, strong noise resistance, and high model accuracy.

We integrated the advantages of SMOTE and XGBboost algorithms to create a new unbalanced classification algorithm named SMOTE-XGBoost.

Details of the method are given below:

Based on the unbalanced dataset, the lesser category has *T* samples, and the SMOTE algorithm will generate the number of new samples as $NT$, where $N$ is a positive integer. Considering the sample $i$ of the lesser category, and its feature vector is ${x}_{i}, i\in \left\{1,\dots ,T\right\}$ , the concrete steps are as follows:

1. *Step 1* Determine the $k$ proximate values of sample ${x}_{i}$ from all $T$ samples of relatively few categories, which can be denoted as ${x}_{inear}, near\in \left\{1,\dots ,k\right\}$.
2. *Step 2* Select a sample ${x}_{i(nn)}$ randomly from the $k$ proximate values. Then we generate a new random number ${\zeta }_{1}$ among 0 ~ 1, and generate a new sample ${x}_{i1}$, as shown in the formula (1).
  1 $${x}_{i1}={x}_{1}+{\zeta }_{1}*({x}_{i(nn)}-{x}_{i})$$
3. *Step 3* Step 2 is repeated $N$ times and then synthesise new $N$ number of samples: ${x}_{inear},new\in 1,\dots N$. Perform the above operation for all *T* minority samples and compose new $NT$ number of samples to generate a new balanced dataset.
4. *Step 4* The newly generated balanced dataset from Step 3 is put into the XGBoost algorithm for product quality prediction.
  The model performance of XGBoost is shown in formula (2):
  2 $${{\widehat{y}}_{i}}^{(m)}=\sum_{j=1}^{m}{f}_{j}\left({x}_{i}\right) \quad {f}_{j} \epsilon F$$
  Formula containing: m is denoted the number of CART models; ${f}_{j}\left({x}_{i}\right)$ is the CART model; ${x}_{i}$ is the $i$th sample of input; ${\widehat{y}}_{i}$ is the $i$th sample of prediction value; $F$ is the collection of all possible CART models.
5. *Step 5* It is not possible to list all possible CARTs at once in tree integration learning algorithms. The XGBoost algorithm uses a gradient boosting strategy that continually repairs previous test results with each new tree added. The prediction value of the $m$ step is ${{\widehat{y}}_{i}}^{(m)}$ ,then the following derivation formula is obtained:
  3 $$\left\{\begin{array}{c}\begin{array}{c}{{\widehat{y}}_{i}}^{(0)}=0\\ {{\widehat{y}}_{i}}^{(1)}={f}_{1}({x}_{i})={{\widehat{y}}_{i}}^{(0)}+{f}_{1}({x}_{i})\\ {{\widehat{y}}_{i}}^{(2)}={f}_{1}({x}_{i})+{f}_{2}({x}_{i})={{\widehat{y}}_{i}}^{(1)}+{f}_{2}({x}_{i})\end{array}\\ \vdots \\ {{\widehat{y}}_{i}}^{(m)}=\sum_{j=1}^{m}{f}_{m}({x}_{i})={{\widehat{y}}_{i}}^{(m-1)}+{f}_{m}({x}_{i})\end{array}\right.$$
  Formula containing: ${{\widehat{y}}_{i}}^{(m)}$ is expressed as the $m$ individual CART optimisation results; ${f}_{m}({x}_{i})$ is denoted the $m$ th CART model.
  Therefore, we obtained the objective function formula (4), which is established by the loss function and the regularisation. The loss function is employed to represent the error between the truth value and the prediction value, and the regularisation term is employed to prevent over-fitting of the model output as shown in formula (5):
  4 $${obj}^{(t)}=\sum_{i=1}^{N}L({y}_{i},{{\widehat{y}}_{i}}^{(t)})+\sum_{j=1}^{m}\Omega ({f}_{j})$$
  5 $$\Omega \left({f}_{j}\right)=\Upsilon T+\frac{1}{2}\lambda {{\omega }_{j}}^{2}$$
  Formula containing: $N$ is denoted the number of samples; $L$ is denoted the loss function; $\Omega \left({f}_{j}\right)$ is the regularisation term of the CART tree, which is used to represent the complexity of the model; $\Upsilon$ is the ${L}_{1}$ penalty parameter that controls the continued splitting of leaf nodes; $\uplambda$ is denoted the penalty parameter of ${L}_{2}$, which prevent the leaf nodes from being overweighted; $T$ is the number of leaf nodes; ${\omega }_{j}$ is the node value.
  The objective function is expanded as in formula (6):
  6 $$ obj^{\left( t \right)} = \Upsilon T + \mathop \sum \limits_{j = 1}^{T} \left[ {\mathop \sum \limits_{{i \in i_{j} }}^{N} L\left( {y_{i} ,\hat{y}_{i}^{{\left( {t - 1} \right)}} + \omega_{j} } \right)} \right] + \frac{1}{2}\lambda \omega_{j}^{2} $$
6. *Step 6* From formula (5), it can be seen that the XGBoost algorithm added $L_{2}$ Regularization term to the complexity term of the tree; that is, $L_{2}$ Smoothing is added for the score of each leaf node. The purpose of this is to avoid over-fitting, and the final objective function is obtained as follows:
  7 $$ obj^{\left( t \right)} = \Upsilon T + \mathop \sum \limits_{j = 1}^{T} \frac{{G_{i}^{2} }}{{H_{i} + \lambda }}\left( {\omega_{j} G_{j} + \frac{1}{2}\omega_{j}^{2} H_{i} + \lambda \omega_{j}^{2} } \right) $$
  $$ G_{i} = \mathop \sum \limits_{{i \in I_{j} }} g_{i} H_{i} = \mathop \sum \limits_{{i \in I_{j} }} h_{i} $$
7. *Step 7* From formula (7), the smaller the value of the objective function, the more optimal it is for the whole tree structure. The final objective function is minimized as follows:
  8 $$ \min_{{obj_{\left( t \right)} }} = \Upsilon T - \frac{1}{2}\mathop \sum \limits_{j = 1}^{T} \frac{{G_{i}^{2} }}{{H_{i} + \lambda }} $$

### SMOTE-XGBoost with hyperparametric optimisation

In the field of classification models, the values of model hyperparameters are frequently disregarded, resulting in the adoption of default settings. This approach may lead to a diminution of model precision. Consequently, it is essential for us to set the critical hyperparameters in the model. The main hyperparametric optimization methods^27^ are the Grid Search algorithm, Random Searching algorithm, Heuristic algorithm, and so on.

Grid search^28^, also known as exhaustive search, initially focuses on identifying and optimizing the parameters that significantly impact the model. Subsequently, it involves systematically traversing all parameters that influence the model within a predetermined range and in a specific sequence until all parameters are optimized. Ultimately, this process results in determining the optimal global combination of parameters. The grid search method supports parallel computing, allowing it to simultaneously evaluate multiple parameter combinations on multiple processors or computing nodes. Therefore, it significantly improves parameter tuning efficiency compared to sequentially performing the evaluation of each set of parameters.

In this article, we employ the grid search method to find the optimization of the primary hyperparameters in the XGBoost classification model. We focus on colsample_bytree, learning_rate, max_depth, and Subsample in XGBoost. Therefore, we have developed an optimal quality monitoring model named SMOTE-XGBoost_s. We deployed the training process of this model in the cloud center and utilized it at the edge computing layer to inspect the quality of assembled products.

### Performance evaluation metrics for unbalanced data

Datasets originating from sample spaces with uneven distributions are referred to as imbalanced datasets. The minority class (non-conforming data) is designated as the positive value, and the majority class (conforming data) as the negative value. The positive values occupy only a minimal portion of the sample space. In traditional model evaluation, Accuracy (ACC) and Equal Error Rate (EER) are usually used as evaluation metrics. However, when the samples show an unbalanced distribution, even if the recognition rate for the minority classes is low, the impact on the whole recognition accuracy is small, and a relatively high accuracy rate can still be obtained. Therefore, when dealing with imbalanced datasets, relying solely on accuracy as an evaluation metric does not accurately reflect the classifier’s performance. In order to address the weaknesses of traditional evaluation metrics, it is imperative to adopt evaluation metrics that can provide more information. Before introducing these evaluation metrics, this article introduces the four foundational concepts of Confusion Matrix, as indicated in Table 1.

**Table 1:** Confusion matrix.

	Positive forecast	Negative forecast
True positive	TP (True positive)	FN (False negative)
True negative	FP (False positive)	TN (True negative)

True Positive (TP): Predicting true positive values as positive values means that true failed samples are predicted as failed.
True Negative (TN): Predicting true negative values as negative values means that true passed samples are predicted as passed.
False Positive (FP): Predicting true negative values as positive values means that true passed samples are predicted as failed.
False Negative (FN): Predicting true positive values as negative values means that true failed samples are predicted as passed.

The confusion matrix is employed to evaluate the function of the algorithm, and five evaluation metrics are selected as the performance evaluation criteria to measure unbalanced data learning in this article.

1. Accuracy (ACC)
  Accuracy represents the probability of all correctly classified samples and measures the accuracy of the classification model. The value range of ACC is [0,1], the larger the value, the better the classification performance, and the formulae is as following:
  9 $$ACC=\frac{TP+TN}{TP+FP+TN+FN}$$
2. Precision (P)
  Precision enables the measurement of the accuracy of the classification of the positive class samples. Among the samples with positive true classification, it indicated the proportion of samples with positive classification predictions, and the formulae is as following:
  10 $$precision=\frac{TP}{TP+FP}$$
3. Recall (R)
  Recall is a measure of the completeness of the classifier, which indicates the proportion of samples with positive true category values that the classifier predicts into positive values, and the formulae is as following:
  11 $$recall=\frac{TP}{TP+FN}$$
4. Geometric mean (G-mean)
  G-mean provides a better measure of the classifier’s ability to classify positive and negative class samples, and the value range of G-mean is [0,1]. The larger the G-mean value, the higher the classification balance ability of the classifier, and the formulae is as following:
  12 $$G-mean=\sqrt{\frac{TN}{(TN+FP)}\cdot \frac{TP}{(TP+FN)}}$$
5. Matthews correlation coefficient (MCC)
  The Matthews correlation coefficient is a more balanced evaluation metric often employed to calculate the correlation between the actual and predicted samples. The value of [− 1,1] and the value of 1 mean perfect prediction. The value of 0 means that the predicted results are not as good as the results of the random prediction, and the value of − 1 means that the predicted classifications do not match the actual classifications at all and the calculation formula is as following:
  13 $$MCC=\frac{TP\times TN-FP\times FN}{\sqrt{(TP+FP)(TP+FN)(TN+FP)(TN+FN)}}$$

## Case study

The article takes the IGBT module bolt assembly production line as a case study. The experimental objects are the IGBT module and M6*8 bolt group. The tightening equipment sensor records the data in real-time. The tightening data of the IGBT module bolt assembly has been determined through experimental analysis, leading to the final experimental conclusion.

### Experimental background

In the discrete manufacturing process, bolted connections are everywhere, and failures in the assembly process occur mainly in the fasteners, which account for about 70% of the failures. Plenty of bolt connections are located on the assembly line of IGBT modules for the traction system. The torque values and angle values of the bolts are crucial for ensuring the safety and reliability of the product structure, as they have an impact on the device’s static and dynamic characteristics, vibration resistance, and other significant performance indicators. Therefore, it is essential to monitor the bolt assembly line. The effective control of IGBT product quality relies on accurate monitoring of the screwing process data in the bolt assembly line.

**Figure 5:**
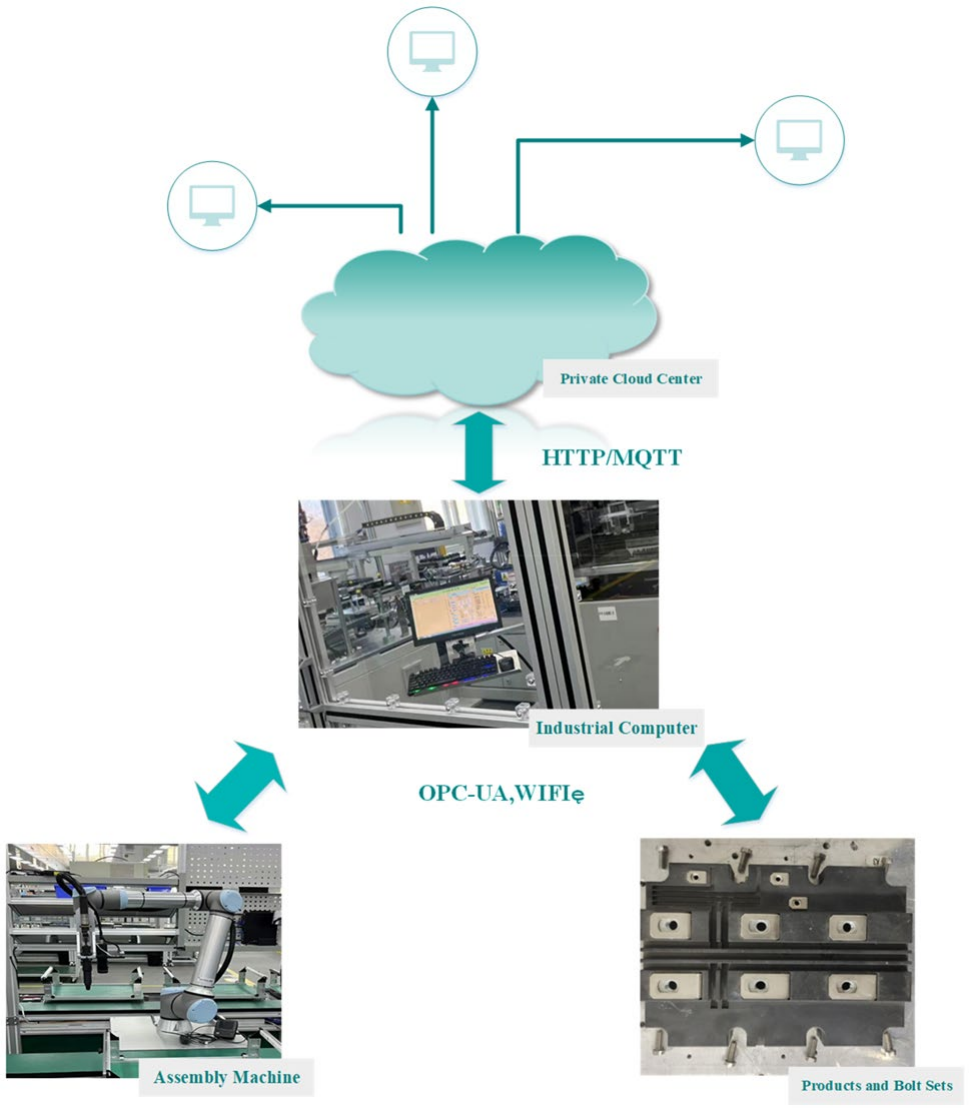
Bolt screwing framework based on industrial IoT of edge computing.

We propose a bolt-screwing framework based on industrial IoT of edge computing, as shown in Fig. 5. The production line is equipped with in-line screwing equipment and an industrial control machine. The in-line screwing equipment is equipped to collect torque information in real-time and then monitor the quality of the product in real-time. Each in-line screwing device will be connected to the industrial controller of the edge device of the production line through OPC-UA, WIFI, and other means. The industrial controller device has the function of storing and processing the collected data. The device is capable of receiving the periodically updated model from the cloud using the HTTP/MQTT protocol. The in-line screwing equipment transmits unlabeled data in real-time through OPC-UA to the industrial PC located at the edge-side. The industrial PC then carries out pre-processing and feature engineering. Then, the assembly of IGBT products is assessed using a quality monitoring model. Finally, the data from the real-time sensors and their corresponding results are uploaded to the private cloud center during periods of network inactivity.

In order to assess the efficacy of the assembly quality monitoring method proposed in this article, real-time samples and records of torque and angle values were collected from the bolt tightening machine in the IGBT module assembly line. These data were utilized as experimental data. The data structure revealed that all sampled data represent qualified products post-process setup monitoring. However, process anomalies were observed in qualified samples from a specific batch of bolts. Upon closer inspection, it was found that despite matching the process settings, variations in part quality and material strength across different batches contributed to unusual data patterns.

The scale of the samples selected for the experiment and the number of normal and abnormal samples are shown in Table 2. The total number of samples in the dataset is 5053, the number of normal samples is 4902, the number of abnormal samples is 151, and the proportion of abnormal samples is 2.9%, which is an unbalanced data learning problem. The data in Table 2 is divided into a training set and a test set, where the training set is 70% and the test set is 30%.

**Table 2:** Description of sample data.

Total	Normal sample size	Number of abnormal samples	Percentage of abnormal samples
5053	4902	151	2.9%

### Experiments and results

Utilizing the bolt screwing data obtained from the IGBT module of the traction system as the primary data source, the LightGBM algorithm is employed to assess the significance of each quality feature and arrange them in a specific order. This process aims to effectively showcase the performance of the prediction model, as shown in Fig. 5. Finally, the final quality characteristics are obtained, as shown in the Table 3.

**Table 3:** Description of features after ranking of importance.

Seriation	Feature name	Significant
1	Feature 5	Torque value
2	Feature 6	Angle value
3	Feature 1	Part number
4	Minute	Time/minute
5	Feature 4	Tighten standards
6	Feature 3	Workstation number
7	Feature 12	Batch number
8	Feature 10	Tightening time
9	Day	Time/day
10	Hour	Time/hour
11	Feature 2	Date code

**Figure 6:**
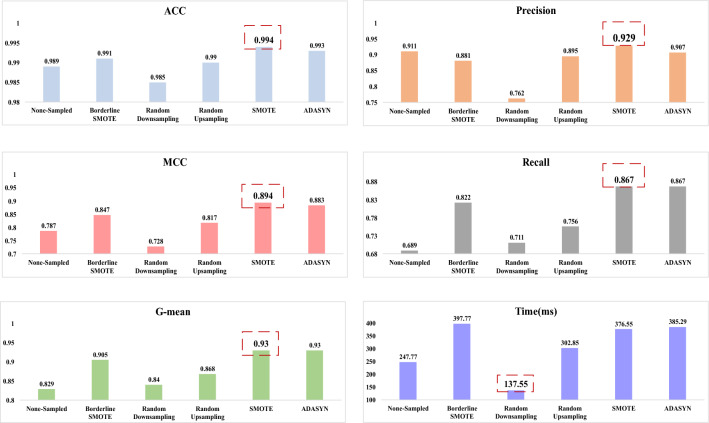
The comparision of evaluation criteriaes and duration of different sampling methods.

In order to comprehensively evaluate the overall performance of the sampling algorithms, we selected five different sampling algorithms for comparative study based on the specified evaluation metrics, including Borderline SMOTE, random downsampling, random upsampling, SMOTE, and ADASYN. A series of comparative experiments were conducted by combining these sampling algorithms in conjunction with the XGBoost classifier. The experimental results are displayed in Fig. 6. The SMOTE sampling method shows superior results in this comparison.

**Figure 7:**
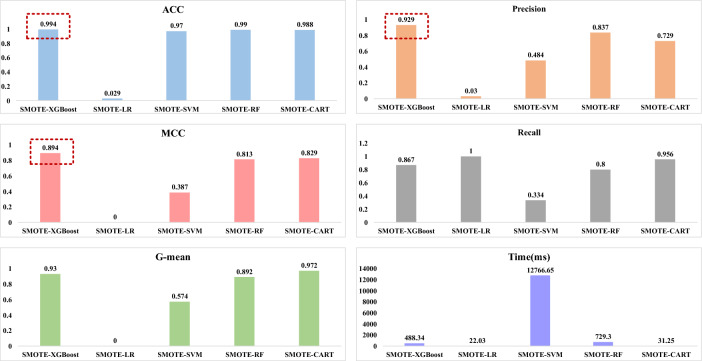
The comparision of evaluation criteriaes and duration of different classification methods.

The quality monitoring model is built using the training set. Meanwhile, in order to validate the classification performance of different classification methods under the same criterion, the SMOTE sampling algorithm combined with EXtreme Gradient Boosting (XGBoost), Support Vector Machine (SVM), Linear Regression (LR), Classification and Regression Tree (CART), and Random Forest (RF) commonly used classification methods are compared. As shown in Fig. 7, the SMOTE-XGBoost quality monitoring model is more effective.

In optimizing the hyperparameters of the SMOTE-XGBoost quality monitoring model, grid search and random search methods were employed to determine the values of the SMOTE-XGBoost hyperparameters, as shown in the Table 4. During this process, the model optimized using the grid search method was named SMOTE-XGBoost_s, while the model optimized using the random search method was referred to as SMOTE-XGBoost_r. Based on the evaluation criteria, the comparison was made between the models before and after optimization. The comparison results, presented in the Table 5, indicate that the SMOTE-XGBoost_s model demonstrated superior performance.

**Table 4:** Optimised values of model hyperparameters.

Parameter name	Default parameter	GS optimal parameter	RS optimal parameter
learning_rate	0.3	0.9	0.15
max_depth	6	12	8
subsample	1	0.9	0.7
colsample_bytree	1	0.5	0.6

**Table 5:** Influence of hyperparameters optimization in models.

Trainning model	ACC	Precision	Recall	MCC	G-mean	Time (ms)
SMOTE-XGBoost	0.994	0.929	0.867	0.894	0.93	488.34
SMOTE-XGBoost_r	0.988	0.903	0.913	0.800	0.898	246.28
SMOTE-XGBoost_s	0.995	0.911	0.911	0.908	0.953	341.21

### Discussion

From the perspective of data, it is essential to select appropriate features to accurately predict the quality of the product and analyze the quality of the product. During the assembly process, there are certainly some unnecessary or less relevant quality features with the final quality. In order to select the most appropriate quality features, we use the LGBM algorithm to calculate the importance of each feature, as shown in Fig. 4. The final quality features are filtered according to the importance of each feature, as shown in Table 3.

From the perspective of the unbalanced data, the Fig. 6 indicates the comparative effects of Unsampled, Borderline SMOTE, Random Downsampling, Random Upsampling, SMOTE, and ADASYN. Figure 7 illustrates the incorporation of SMOTE in conjunction with various classification algorithms, namely XGBoost, SVM, Logistic regression, Decision Tree, and Random Forest. Table 4 indicates the optimal parameters of the hyperparameters finally determined after the hyperparameter optimization performed by SMOTE-XGBoost. Table 5 shows that the SMOTE-XGBoost method with hyperparameter optimisation improves the classification performance. The above demonstrates that the method proposed in this article has a positive impact on the issue of classifying unbalanced data. In mainstream classification learning, accuracy is the most frequently employed criterion for evaluation. However, the various conventional evaluation standards used to assess classifier performance are not applicable in unbalanced datasets. Therefore, we selected five metrics: Accuracy, Precision, Recall, G-mean, and MCC. MCC and G-mean are used as comprehensive metrics, considering both qualified and faulty products. In the context of unbalanced assembly quality monitoring, accuracy, MCC, and G-mean are considered to be more crucial metrics compared to other evaluation criteria. These metrics are extensively employed in diverse unbalanced classification problems.

## Conclusion

This study proposes a framework for monitoring product assembly quality based on edge computing, with the aim of addressing the issue of data imbalance in the assembly process. Firstly, the real-time data collected at the edge of the production line is preprocessed. For the feature engineering, the LGBM algorithm should be utilized to conduct feature ranking and feature selection. Moreover, we select the most appropriate sampling method based on the evaluation criteria, namly SMOTE algorithm. Finally, we propose a SMOTE-XGBoost_s monitoring model based on hyperparameter optimization to solve the quality monitoring problem in the case of unbalanced data.

The experimental results presented in this study demonstrate the superior adaptability of the proposed SMOTE sampling technique compared to alternative sampling methods. The SMOTE-XGBoost method is created by integrating the SMOTE algorithm with XGBoost algorithm. This method has been found to outperform the combination of the SMOTE algorithm with other classification algorithms. Next, the hyperparameters of the XGBoost algorithm in the SMOTE-XGBoost model were optimized using both grid search and random search algorithms. Through experimental comparison, the model performance after optimization using the grid search algorithm is better. Consequently, the optimized model was designated as SMOTE-XGBoost_s. The above content indicates that the method proposed in this article has significant value in product quality control and unbalanced data processing. It confirms the validity and applicability of the method in practical applications.

It has been experimentally demonstrated that the SMOTE-XGBoost_s method, as proposed in this paper, is highly effective in monitoring the quality of product assembly. The research results of this paper will support the improvement in the product quality monitoring technology of manufacturing industry and provide intelligent information service for factory managers. However, there are still problems that need to be further researched. The proposed method in this article was applied in the edge computing scenario. It failed to consider that the amount of data during the assembly process would continuously increase. Therefore, the next issue that needs to be carefully explored is the degree of match between the quality monitoring model and the incremental data.

## Data Availability

The datasets generated during and/or analysed during the current study are not publicly available due to [Information related to product processing] but are available from the corresponding author on reasonable request.

## References

[1] Qiao HH, Wang TY, Wang P (2020). A tool wear monitoring and prediction system based on multiscale deep learning models and fog computing. Int. J. Adv. Manuf. Technol..

[2] Shi W, Cao J, Zhang Q (2016). Edge computing: Vision and challenges. IEEE Intern. Things J..

[3] Campolo, C., Genovese, G., Molinaro, A. *et al.* Digital twins at the edge to track mobility for maas applications. In: *Proceedings of the IEEE/ACM 24th International Symposium on Distributed Simulation and Real Time Applications (DS-RT), Electr Network, Sep 14–16, 2020* (2020).

[4] Adhikari M, Munusamy A, Hazra A (2022). Security in edge-centric intelligent internet of vehicles: Issues and remedies. IEEE Consum. Electron. Mag..

[5] Wang HX, Liu TT, Kim B (2020). Architectural design alternatives based on cloud/edge/fog computing for connected vehicles. IEEE Commun. Surv. Tutor..

[6] Qin, B. L., Luo, Q., Luo, Y. S. *et al.* Research and application of key technologies of edge computing for industrial robots. In: *Proceedings of the 4th IEEE Information Technology, Networking, Electronic and Automation Control Conference (ITNEC), Electr Network, Jun 12–14, 2020* (2020).

[7] Qiu T, Chi JC, Zhou XB (2020). Edge computing in industrial internet of things: Architecture, advances and challenges. IEEE Commun. Surv. Tutor..

[8] Tian YL, Li T, Xiong JB (2022). A blockchain-based machine learning framework for edge services in iiot. IEEE Trans. Ind. Inf..

[9] Usman M, Jolfaei A, Jan MA (2020). Rasec: An intelligent framework for reliable and secure multilevel edge computing in industrial environments. IEEE Trans. Ind. Appl..

[10] Zhao Y, Ye H (2023). Power system low delay resource scheduling model based on edge computing node. Sci. Rep..

[11] Gang Q, Siliang L, Donghui P (2019). Edge computing: A promising framework for real-time fault diagnosis and dynamic control of rotating machines using multi-sensor data. IEEE Sens. J..

[12] Blanco-Novoa O, Fernandez-Carames TM, Fraga-Lamas P, Vilar-Montesinos MA (2018). A practical evaluation of commercial industrial augmented reality systems in an industry 4.0 shipyard. IEEE Access.

[13] Atan B, Basaran M, Calik N (2023). Ai-empowered fast task execution decision for delay-sensitive iot applications in edge computing networks. IEEE Access.

[14] Fan, L., Wei, Y., Xing, L. *et al.* Toward edge-based deep learning in industrial Internet of Things. *IEEE Intern. Things J.* 1 (2020). 10.1109/jiot.2019.2963635 PMC10938735 38486787

[15] Wang T, Ke HX, Zheng X (2020). Big data cleaning based on mobile edge computing in industrial sensor-cloud. IEEE Trans. Ind. Inf..

[16] Yu J, Xi L-f (2008). Intelligent monitoring and diagnosis of manufacturing process using an integrated approach of neural network ensemble and genetic algorithm. Int. J. Comput. Appl. Technol..

[17] Yang KS, Zhao LY, Wang CL (2022). A new intelligent bearing fault diagnosis model based on triplet network and svm. Sci. Rep..

[18] Rowlands H, Wang LR (2000). An approach of fuzzy logic evaluation and control in spc. Qual. Reliab. Eng. Int..

[19] You DY, Gao XD, Katayama S (2015). Wpd-pca-based laser welding process monitoring and defects diagnosis by using fnn and svm. IEEE Trans. Ind. Electron..

[20] Chen W, Zou B, Li YS (2021). A study of a rapid method for detecting the machined surface roughness. Int. J. Adv. Manuf. Technol..

[21] Ren L, Meng ZH, Wang XK (2020). A wide-deep-sequence model-based quality prediction method in industrial process analysis. IEEE Trans. Neural Netw. Learn. Syst..

[22] Zhou P, Jiang Y, Wen CY (2019). Data modeling for quality prediction using improved orthogonal incremental random vector functional-link networks. Neurocomputing.

[23] Zhang CF, Dong J, Peng KX (2023). A novel quality-related process monitoring method for multi-unit industrial processes under incomplete data conditions. Can. J. Chem. Eng..

[24] Wang L, Han M, Li XJ (2021). Review of classification methods on unbalanced data sets. IEEE Access.

[25] Chen, T. & Guestrin, C. Xgboost: A scalable tree boosting system. In *CoRR* (2016). arXiv:1603.02754

[26] Hancock JT, Khoshgoftaar TM (2020). Catboost for big data: An interdisciplinary review. J. Big Data.

[27] Bergstra J, Bengio Y (2012). Random search for hyper-parameter optimization. J. Mach. Learn. Res..

[28] Wang, Q., Wang, P. H., Su, Z. G. *et al.* A hybrid search strategy based particle swarm optimization algorithm. In: *Proceedings of the 8th IEEE Conference on Industrial Electronics and Applications (ICIEA), Swinburne Univ Technol, Melbourne, Australia, Jun 19–21, 2013* (2013).

